# Text-Book of Animal Physiology

**Published:** 1890-01

**Authors:** 


"Text-Book of Animal Physiology." By Wesley Mills, A, M., M. D., L. R. C. P. (Eng.), Professor of Physiology in McGill University and the Veterinary College, Montreal. Published by. D. Appleton & Co., N. Y.. For sale by Richards & Son, Atlanta, Ga.

As science progresses the uneducated, who pose as doctors and veterinarians, must seek new fields or be relegated to the back-ground. This book is a step in the right direction, and while perhaps a little too deep for students and general practitioners, they will in time grow up to it. It is, in fact, a step in advance of the times, but is a book that will well repay the reading. F. O. S.

